# Clinical support and perceived competency levels of midwifery students: A descriptive analysis

**DOI:** 10.4102/hsag.v27i0.1783

**Published:** 2022-11-03

**Authors:** Hafaza B. Amod, Sipho W. Mkhize

**Affiliations:** 1 Department of Nursing, Faculty of Nursing and Public Health, University of KwaZulu-Natal, Durban, South Africa

**Keywords:** clinical supervision, clinical support, mentorship, midwifery students, perceived competency levels

## Abstract

**Background:**

Midwifery students in South Africa place great value on the clinical support they receive from midwifery practitioners. Adequate clinical support should help midwifery students to practice procedures safely and independently, allowing them to be competent upon degree completion.

**Aim:**

To describe the clinical support and perceived competency levels of midwifery students.

**Setting:**

Public hospitals in KwaZulu-Natal.

**Methods:**

The researcher chose a quantitative research method using a descriptive design. An all-inclusive purposive and convenience sampling method was undertaken to recruit midwifery students from an undergraduate nursing programme at a university in KwaZulu-Natal. Gatekeepers permission and ethics approval was obtained from the university’s registrar and research ethics committee. A self-evaluation questionnaire describing the clinical support and perceived competency levels was completed by 60 respondents. Data were analysed using International Business Machines Corporation Statistical Package for Social Sciences (IBM-SPSS) Version 27.

**Results:**

The results highlighted that the clinical support midwifery students received, was beneficial to their clinical learning outcomes. Eighty per cent of clinical support offered to midwifery students was obtained through clinical supervision. Ninety-three per cent of respondents revealed that the clinical support they received were from midwifery practitioners (without a speciality qualification). Although students rated themselves as competent in 88.6% of midwifery procedures, poor outcomes were identified in 11.4% procedures.

**Conclusion:**

Midwifery practitioners play a significant role in supporting midwifery students during clinical placement. Advancing the roles of midwifery practitioners through mentorship training is likely to strengthen the quality of clinical support provided and thus improve the competence levels of midwifery students.

**Contribution:**

The findings in this paper are valuable in developing clinical support training guidelines for midwifery practitioners.

## Background

In South Africa, the quality of maternity care and its contribution to maternal mortality remains a significant challenge. According to the most recent Saving Mothers Report (2016–2018), the general lack of knowledge and skills of maternity care providers accounted for 25% of all avoidable maternal deaths. In response, the Department of Health-South Africa (DoH-SA) recommended that undergraduate training levels of healthcare practitioners’ knowledge and skills should be improved (Saving Mothers Report 2016–2018).

Students in the midwifery module of the R425 programme are required to complete a minimum of 1000 clinical hours (South African Nursing Council [SANC], R425 of 22 February 1985). As a result, midwifery students spend the majority of their module time in clinical placements working in antenatal, labor, postnatal, and newborn units on a rotational basis to meet these high expectations. Clinical placement in maternity wards can be intensely challenging for midwifery students in terms of the labour ward demands and organizational tensions (Coldridge & Davies 2017); the effecting nurse teacher, and the educational atmosphere (Arkan, Ordin & Yilmaz 2018); and high student intakes and disorganized learning opportunities (McKellar & Graham 2017; Rahimi et al. 2019). In South African public hospitals, high patient turnover and staff shortages, offers midwifery students many opportunities to manage maternity cases with diverse health care needs (Matlala & Lumadi 2019; Thopola & Lekhuleni 2015).

Students place great value on the support and experience they receive from midwifery practitioners during clinical placement (Power & Grzelak 2016). According to Thunes and Sekse (2015), the clinical support received during maternity placement is profoundly fundamental to the development of midwifery students. In South Africa, midwifery practitioners who support students in these clinical placements do not receive any formal training or support, and therefore, their supportive role is primarily voluntary. More so, midwifery practitioners often find themselves juggling between patient care priorities and student supervision (Maputle, Malwela & Lebese 2016). When midwifery students are competent in all clinical requirements then midwifery lecturers have reason to believe that the clinical learning and support that students received, was adequate (Russell, Alliex & Gluyas 2016). On the contrary, when students remain incompetent, then lecturers need to reassess the clinical support that students received during clinical placements.

Poor clinical outcomes were evident in a study done by Yigzaw et al. (2015), which revealed that the overall average competence score for midwifery students on point of graduation was 51.8%. In another study, poor clinical supervision of midwifery students revealed competency scores less than 50% in all clinical skills (Malakooti, Bahadoran & Ehsaanpoor 2020). Hence, the effects of poor clinical support may have negative connotations on the clinical competence of midwifery students. It is therefore important that midwifery educators and managers constantly monitor and improve the current clinical support offered to midwifery students during their clinical placement.

### Aim

The aim of this article is to describe the clinical support provided to midwifery students and their perceived competency levels in midwifery requirements post clinical placement at public hospitals in KwaZulu-Natal.

### Research objective

To analyse the current clinical support and the perceived competency levels of undergraduate midwifery students from a selected higher education institution in South Africa.

## Research method

This study is part of a larger study on analysing and strengthening the clinical support of undergraduate midwifery students and developing a mentorship training programme at a higher education institution in KwaZulu-Natal: A mixed method and action research design. This article is step 2 of cycle 1 which employed a quantitative approach with a descriptive research design to describe the clinical support and the perceived competency levels of midwifery students, following clinical placement at five public hospitals.

### Research approach and design

Descriptive research is quantitative in nature as it attempts to collect numerical data and statistically analyse it to describe and explain the variables, situation or a phenomenon (Bloomfield & Fisher 2019). The researchers used a descriptive research design to analyse the clinical experience and the perceived competency levels of midwifery students from the findings of a self-evaluation questionnaire.

### Research setting

This study was conducted at a higher education institution in South Africa. Students enrolled into the baccalaureate of nursing programme are expected to complete a module in midwifery practice in the fourth year of training. The university is centrally located within the same local district as the five public hospitals where undergraduate midwifery students are placed for clinical learning.

### Population

All fourth year students enrolled for the midwifery module in an undergraduate nursing programme offered at the university.

### Sample and sampling

The sampling technique was aligned to the aim of the study which was to describe the clinical support and perceived competency levels of midwifery students. An all-inclusive purposive and convenient sampling method was undertaken to recruit midwifery students from an undergraduate nursing programme, at a university in KwaZulu-Natal. The method was purposive as the study used a specific cohort from the midwifery class to analyse the clinical support of midwifery students from a higher education institution. The method was also convenient as data was collected from students when they returned to campus post-clinical placement in five public hospitals. A sample size of all 68 students were selected for this survey.

### Research tool

A self-evaluation questionnaire was used to collect data. The questionnaire was developed by the researcher and the research supervisor to describe the clinical support of students and assess their competency levels. The researchers adapted the tool from the works of Hogan, Fox and Barratt-See (2017). The design of the research questionnaire was aligned to the International Confederation of Midwives (ICM 2019) and the SANC (2013) competencies for midwifery clinical practice. The questionnaire comprised three sections which included the demographic profiles; the clinical support and students’ perceived levels of competencies. Questionnaire items were close-ended and hence restricted respondents to select only specific choices. The results were therefore contained within expected choices.

### Data collection process

For this study, data was collected on campus from midwifery students who completed their clinical placements in maternity departments at five public hospitals in KwaZulu-Natal. Prior to data collection, the researchers briefed students on the purpose and expectations of the study. Each recruited student received an information sheet and then signed the informed consent form. Data collection took place during the month of February 2021. All completed questionnaires were hand-collected, captured electronically and safely stored by the researchers. Out of 68 students in the cohort, only 60 students participated in this study. A response rate of 88% was achieved.

### Data analysis

Data were saved in an excel spreadsheet and analysed in a computer-based software package for social sciences called International Business Machines Corporation Statistical Package for Social Sciences (IBM-SPSS) Version 27 (2020). All data were imported to SPSS and analysed using descriptive statistics.

### Reliability and validity

The reliability of the questionnaire was established in a previous study by Hogan et al. (2017); however, the researchers adapted the tool and developed questionnaire items to suit the objectives of this study. The validity of the questionnaire was pre-tested with a small group of eight students from the previous cohort of midwives. The Cronbach’s Alpha score of > 0.7 is considered to be a good reliability score and a score of 0.9 was obtained for this questionnaire items.

### Ethical considerations

This study was ethically reviewed and approved by the University of KwaZulu-Natal Humanities and Social Sciences Research Ethics Commitee (approval number: HSS/1509/018M). The confidentiality, anonymity and respect for respondents were maintained throughout the study.

## Findings

The results of this study are presented according to the design of the research questionnaire and is presented in sections A, B and C respectively. Section A describes the demographic profiles of respondents, Section B describes the clinical support that midwifery students received during their clinical placement, and Section C describes how midwifery students perceived their competency levels post clinical placement. The data captured in Section C was aligned to the ICM competencies for midwifery practice and included four areas of care namely the general care, the pre-and antenatal care, the labour care and the postnatal care. The midwifery requirements are the expected minimum requirements for undergraduate midwifery students prescribed by the SANC (SANC, R425 of 22 February 1985) and the university college handbook, (University of KwaZulu-Natal College of Health Science 2021).

### Section A: Demographic profiles

The results of this section measured categorical data and are reflected in frequencies and percentages.

#### Age and gender profiles

There was a total of 43 females and 17 males in the study. Eighty seven per cent of respondents (*n* = 52) were between the ages of 18 and 24 years of which 39 were females and 13 males. The remaining eight respondents accounted for the 13% of the study population which comprised four males and four females over the age of 25 years.

#### Country/province of origin

At the time of this study, all respondents (*N* = 60) were currently residing in three different provinces within South Africa. Eighty seven per cent of respondents (*n* = 52) live within the KwaZulu-Natal province whilst 10% (*n* = 6) respondents live in the Eastern Cape, and 3% (*n* = 2) in Mpumalanga province.

#### Clinical facilities

All respondents (*N* = 60) were allocated at five public hospitals within the eThekwini district, for experiential learning. Two of these hospitals (Institutions A and B) are classified as district hospitals. In Institution A, there were 11 (18.3%) respondents and in Institution B there were 9 (15%) respondents. Three hospitals (Institutions C, D, and E) are regional hospitals. The highest number of respondents, which was 16 (26.6%) were placed at Institution D, followed by 14 (23%) respondents placed at Institution E. The least number of respondents, which was 10 (16.6%) were placed in Institution C.

#### Person responsible for clinical support in public hospital

**FIGURE 1:**
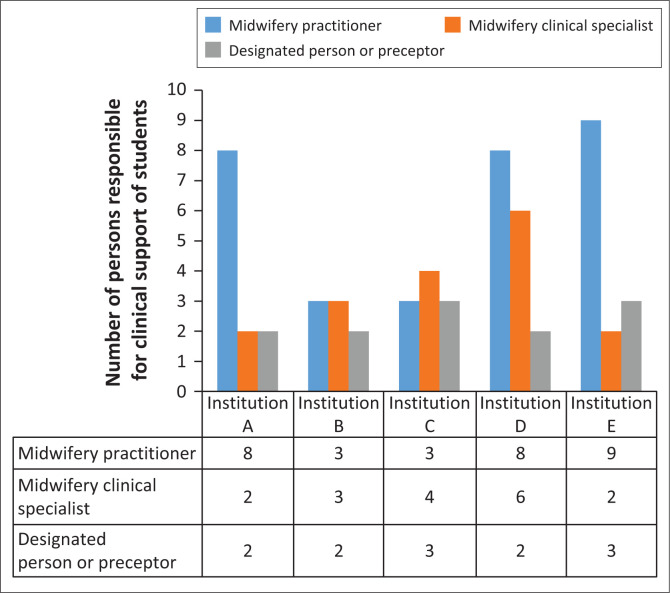
Person responsible for clinical support of students.

The results relating to the clinical support that students received during clinical placement indicated that 51.7% (*n* = 31) was received from midwifery practitioners who did not have a speciality qualification followed by 28.3% (*n* = 17) of midwifery clinical specialists, and lastly 20% (*n* = 12) by a designated person who is the assigned preceptor for student supervision during clinical placement. The results also highlighted that most clinical support (*n* = 9) were received from midwifery practitioners working in Institution E; and this was closely followed in institutions D (*n* = 8) and A (*n* = 8). These results are seen in Figure 1.

### Section B: Clinical support received at clinical facilities

This section describes the clinical support received by midwifery students during clinical placement for antenatal care; labour care and postnatal care. It also identifies how clinical placement contributed to midwifery students’ learning outcomes. The results are presented in frequencies and percentages.

#### Type of clinical support

**FIGURE 2:**
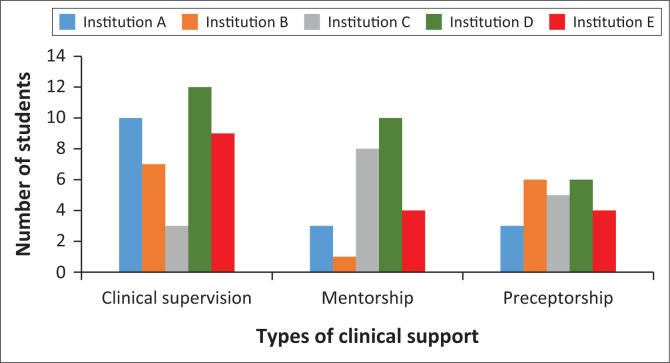
Types of clinical support across the different clinical facilities.

Figure 2 displays the three frequent types of clinical support offered across the five public hospitals. The findings show that clinical supervision (*n* = 40) was highly practiced across all clinical placements, followed by mentorship (*n* = 26) and then preceptorship (*n* = 24). However, Institution B fared extremely poorly (*n* = 1) in mentorship.

All respondents (*n* = 60) confirmed that they received some type of clinical support during their placement, either to a greater or lesser extent. As seen in Table 1, 80% (*n* = 48) of the clinical support was through clinical supervision, followed by mentorship (51.6%; *n* = 31) and lastly preceptorship (46.6%; *n* = 728). Eighty per cent of clinical supervision (*n* = 48) occurred during labour ward placement, followed by antenatal ward (68.3%; *n* = 41) and then postnatal ward (66.7%; *n* = 40).

**TABLE 1:** Type of clinical support received during placement in different maternity care areas.

Types of clinical support	Antenatal care	Labour care	Postnatal care
**Clinical supervision**			
Greater extent	41	48	40
Lesser extent	19	12	20
**Total**	**60**	**60**	**60**
**Preceptorship**			
Greater extent	26	28	24
Lesser extent	34	32	36
**Total**	**60**	**60**	**60**
**Mentorship**			
Greater extent	24	27	31
Lesser extent	36	33	29
**Total**	**60**	**60**	**60**

#### The benefit of clinical support to learning

**FIGURE 3:**
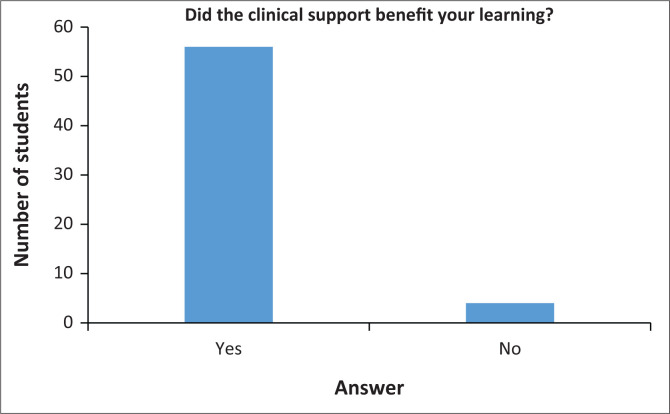
The benefit of clinical learning.

Clinical placement aims to ensure that students integrate theoretical knowledge with practical experiences. Fifty-six respondents (93%) reported that the overall clinical support they received was beneficial to their clinical learning as they had achieved the minimum set of midwifery requirements whilst four respondents (7%) had disagreed with the statement. Figure 3 presents these results.

### Section C: Results of students’ perceived levels of competence at the end of the module

Section C describes how midwifery students perceived their levels of competence in clinical requirements for the undergraduate nursing degree. This section has four categories aligned to the ICM essential competencies for midwifery clinical practice namely: (1) general care, (2) pre-antenatal care, (3) labour care, and (4) postnatal care (including newborn). The general care category includes general requirements that students are expected to acquire throughout their degree programme and these general requirements are used across categories 2, 3 and 4. A Likert scale measured the levels of competence in a total of 70 midwifery requirements as follows: The results highlighted that respondents were competent in 88.6% of the total requirements for midwifery clinical practice and were incompetent in 11.4% of the requirements. Tables 2–5 highlight the results of perceived competency levels in each category.

**TABLE 2:** General care requirements.

General care requirements	0 = Incompetent	1 = Competent	Mean	Median	Standard deviation	Valid
Communicate using effective interpersonal skills	16	44	2.73	3.00	0.446	60
Demonstrate good clinical judgement and reasoning	18	42	2.70	3.00	0.462	60
Work effectively in a team	11	49	2.82	3.00	0.390	60
Practice professional conduct	12	48	2.80	3.00	0.403	60
Accept responsibility for acts and omissions	14	46	2.77	3.00	0.427	60
Maintain effective writing skills and complete documentation	20	40	2.67	3.00	0.475	60
Demonstrate cultural awareness and sensitivity	17	43	2.70	3.00	0.497	60

**TABLE 3:** Pre-antenatal care requirements.

Pre-antenatal requirements	0	1	Mean	Median	Standard deviation	Valid
Identify the signs and symptoms of pregnancy	13	47	2.75	3.00	0.508	60
Conduct prenatal and antenatal history taking	20	40	2.63	3.00	0.551	60
Conduct a full physical examination of a pregnant woman	16	44	2.72	3.00	0.490	60
Conduct an abdominal examination of a pregnant woman	11	49	2.82	3.00	0.390	60
Calculate the EDD using Naegele’s rule	8	52	2.83	3.00	0.457	60
Perform a pap smear	47	13	1.68	1.00	0.813	60
Recognise the minor and common disorders in pregnancy	22	42	2.55	3.00	0.649	60
Give advice on the common disorders in pregnancy	18	38	2.68	3.00	0.504	60
Teach antenatal exercises	13	47	2.73	3.00	0.548	60
Monitor and record FKC	13	47	2.78	3.00	0.415	60
Perform a pelvic assessment to detect abnormalities	45	15	2.03	2.00	0.688	60
Give appropriate health education	15	45	2.75	3.00	0.437	60
Screen high-risk pregnancies	33	26	2.38	2.00	0.585	60
Identify abnormal changes during pregnancy	27	33	2.50	3.00	0.597	60
Formulate a nursing care plan for identified needs	20	40	2.62	3.00	0.585	60

EDD, expected date of delivery; FKC, fetal kick counts.

**TABLE 4:** Labour care requirements.

Labour care requirements	0	1	Mean	Median	Standard deviation	Valid
Assess for signs and symptoms of labour	11	49	2.82	3.00	0.390	60
Monitor for contractions – intensity, duration and frequency	19	41	2.68	3.00	0.469	60
Perform a vaginal examination	23	37	2.62	3.00	0.490	60
Confirm the diagnosis of labour	22	38	2.63	3.00	0.486	60
Record data accurately using the partograph	18	42	2.67	3.00	0.542	60
Interpret data accurately using the partograph	19	41	2.68	3.00	0.469	60
Monitor and interpret maternal and fetal condition	16	44	2.72	3.00	0.490	60
Perform artificial rupture of membranes	25	35	2.53	3.00	0.596	60
Monitor a woman on oxytocin infusion	17	43	2.72	3.00	0.454	60
Monitor a woman undergoing an induction of labour	15	45	2.73	3.00	0.482	60
Infiltrate, perform and suture an episiotomy if necessary	35	25	2.35	2.00	0.606	60
Deliver the baby safely following the mechanism of normal labour	9	51	2.85	3.00	0.360	60
Perform passive management of the 3rd stage of labour	14	46	2.70	3.00	0.591	60
Perform active management of the 3rd stage of labour	11	49	2.82	3.00	0.390	60
Examine the perineum and vulva for lacerations	11	49	2.82	3.00	0.390	60
Manage the 4th stage of labour	6	54	2.90	3.00	0.303	60
Check the uterus post delivery	9	51	2.85	3.00	0.360	60
Examine the placenta and membrane	8	52	2.87	3.00	0.343	60
Assess the blood loss	25	35	2.57	3.00	0.533	60

**TABLE 5:** Postnatal care requirements.

Postnatal care requirements	0	1	Mean	Median	Standard deviation	Valid
**Postnatal care of mother**						
Recognise the physiological changes to the reproductive system	33	27	2.42	2.00	0.403	60
Conduct a thorough physical examination post-normal delivery	15	45	2.75	3.00	0.390	60
Conduct a thorough physical examination post-caesarean section	12	48	2.80	3.00	0.303	60
Perform post-delivery breast examination	11	49	2.82	3.00	0.343	60
Monitor symphysis fundal height	6	54	2.90	3.00	0.581	60
Monitor the vaginal discharge/lochia	8	52	2.87	3.00	0.252	60
Perform vulva swabbing	19	41	2.63	3.00	0.415	60
Examine the perineum	4	56	2.93	3.00	0.360	60
Teach the mother the technique of breast feeding	13	47	2.78	3.00	0.533	60
Give relevant health education	9	51	2.85	3.00	0.650	60
Identify problems and potential problems in the care of the mother and baby in the puerperium	25	35	2.57	3.00	0.533	60
Demonstrate post-natal exercise to the women	23	37	2.53	3.00	0.650	60
Counsel on and administer family planning method	9	51	2.85	3.00	0.360	60
Conduct a discharge procedure of a postnatal mother and baby	13	47	2.77	3.00	0.465	60
**Postnatal care of newborn**						
Assess the Apgar score	5	55	2.90	3.00	0.354	60
Complete the immediate care of the newborn	3	57	2.95	3.00	0.220	60
Perform a physical assessment of the neonate	8	52	2.87	3.00	0.343	60
Perform a neurological assessment of the neonate	29	31	2.47	3.00	0.596	60
Perform basic resuscitation on a newborn	46	14	2.02	2.00	0.676	60
Complete birth notification	5	55	2.92	3.00	0.279	60
Transfer a sick neonate to the nursery	25	35	2.53	3.00	0.596	60
Perform first baby bath and teach the mother	33	27	2.18	2.00	0.833	60
Perform cord care and teach the mother	8	52	2.85	3.00	0.404	60
Plan, implement, evaluate the care of the neonate	31	29	2.47	2.00	0.536	60
Administer BCG and polio drops	8	52	2.87	3.00	0.343	60
Care for a baby receiving phototherapy	29	31	2.45	3.00	0.622	60
Perform a stomach washout	43	17	1.88	2.00	0.825	60
Administer a nasogastric feed	27	33	2.42	3.00	0.724	60
Perform dextrose sticks monitoring	25	35	2.42	3.00	0.766	60

BCG, Bacille Calmette-Guerin.

#### Competence in general care

In the general care category, the results expressed that 81.66% (*n* = 49) of respondents work effectively in team and 33.3% (*n* = 20) were not competent in effective writing skills and completion of documentation. Across all clinical requirements, 66.7% (*n* = 40) respondents perceived themselves as competent whilst 33% (*n* = 20) of respondents perceived themselves as incompetent. These results are captured in Table 2. The average mean score in this category was 2.74 which was a score close to the expected average median score of 3.0. A low standard deviation (SD) score of < 0.5 was achieved across all requirements.

#### Competence in pre-antenatal care

In the pre-antenatal care category, there were 86.7% (*n* = 52) respondents competent in calculating the expected date of delivery (EDD) using Naegele’s rule. Forty-nine respondents (81.6%) were competent in conducting an abdominal examination of a pregnant woman and 78.3% (*n* = 47) respondents could identify the signs and symptoms of pregnancy, teach antenatal exercise and monitor and record fetal kick counts (FKC).

High incompetent scores among respondents were evident in obtaining a pap smear (*n* = 47), performing a pelvic assessment (*n* = 45) and screening high risk pregnancies (*n* = 33). The average mean score across competencies was 2.54 and the average median score was 3.0. Low median score < 3.0 were evident in the three poorly perceived midwifery requirements mentioned above. Table 3 reflects these results. All columns reflected in green denotes the antenatal care requirements that students perceived themselves as incompetent in, at the end of the midwifery module.

#### Competence in labour care

Results in labour care revealed that 90% (*n* = 54) of respondents were competent in managing the 4th stage of labour, 88.7% (*n* = 52) were competent in examining the placenta and 85% (*n* = 51) were competent in delivering the baby safely and checking the uterus post-delivery. Thirty-five (58.3%) of respondents were incompetent in infiltrating, performing and suturing of an episiotomy, followed by 42% (*n* = 25) who were incompetent in performing an artificial rupture on membranes and assessing blood loss. The average mean score achieved was 2.71 and the average median score was 3.0 across all requirements as seen in Table 4 below. All columns reflected in green denotes the labour care requirements that students perceived themselves incompetent in, at the end of the midwifery module.

#### Competence in postnatal care

The postnatal care category comprises the care of the postnatal mother and the newborn. In the postnatal mother sub-category, 55% (*n* = 33) respondents scored themselves as incompetent in recognizing the physiological changes to the reproductive system. Ninety-three per cent (*n* = 56) of respondents perceived themselves competent examining the perineum, 90% (*n* = 54) in monitoring the symphysis fundal height and 87% (*n* = 52) in monitoring the vaginal discharge/ lochia, post-delivery.

In the newborn care sub-category, 76.7% (*n* = 46) respondents perceived themselves incompetent in performing basic resuscitation on a newborn. Respondents were also found to be incompetent in performing a stomach washout (71.6%; *n* = 43) and to perform the first baby bath (55%, *n* = 33). These results are visible in Table 5. Lower means scores and corresponding low median scores of 2.0 suggest that respondents needed more opportunities to learn and practice before assessing themselves as competent in all requirements. All columns reflected in green denotes the postnatal care requirements that students perceived themselves incompetent in, at the end of the midwifery module.

**FIGURE 4:**
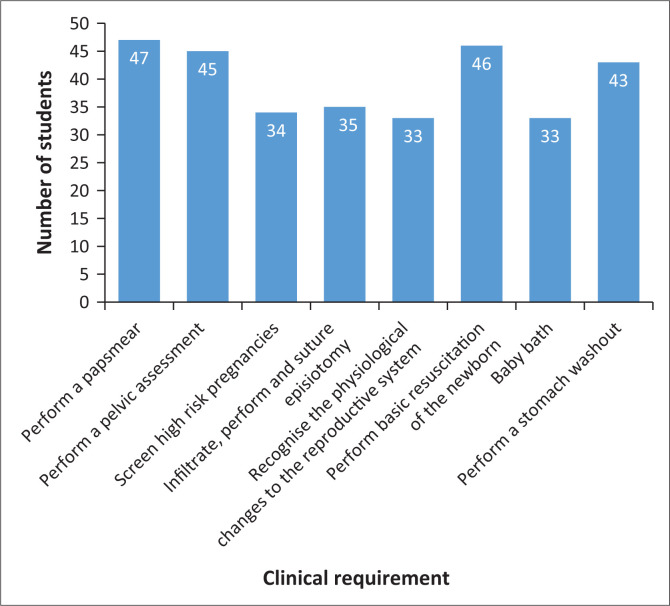
Perceived incompetence in performing clinical requirements.

Figure 4 displays a summary of the requirements that respondents perceived themselves as incompetent in, at the end of their clinical placement. It is noted from these results that a minimum of 55% (*n* = 33) of this cohort were incompetent in these eight midwifery clinical requirements.

## Discussion

The SANC prescribes a minimum of 50% of midwifery clinical hours to be spent in clinical practice for an undergraduate nurse training programme (SANC, R425 of 22 February 1985). The goal is to ensure that midwifery students receive sufficient clinical experience and support to become clinically competent for role-taking upon degree completion. Therefore, achieving competence in all requirements is critical for the transition of a midwifery student into an independent and safe midwifery practitioner (Hussein et al. 2019; Powers, Herron & Pagel 2019).

The benefits of clinical support in nursing are indicated in various mentorship, preceptorship, and clinical supervision approaches globally (John et al. 2020; McSharry & Lathlean 2017; Rambod, Sharif & Khademian 2018; Smith & Sweet 2019; Tuomikoski et al. 2020) and in Africa (Feyissa, Balabanova & Woldie 2019; Lethale, Makhado & Koen 2019). Mentorship in midwifery is fast becoming the acceptable choice of clinical support for midwifery students (Bradshaw, Tighe & Doody 2018; Demirel & Kocatas 2021; Moran & Banks 2016) especially in African countries where improved skills of midwifery practitioners are urgently required to reduce high maternal and neonatal death rates (Musabwasoni et al. 2020; Shikuku et al. 2019).

Clinical supervision and preceptorship also known as ‘bedside teaching’ or using ‘a hands-on approach’ is routinely adopted to supervise midwifery students and newly qualified nurses during clinical placement. In this study, clinical supervision was the most common type of clinical support offered to undergraduate midwifery students, followed by mentorship, and lastly preceptorship. According to Thunes and Sekse (2015) and Sidebotham and Fenwick (2019), midwifery students attribute their clinical success to the midwifery practitioners they work with on a daily basis. In this study, respondents shared the same sentiments where 93% of midwifery students found the clinical support that they received from registered midwives across all five public hospitals was beneficial to their clinical learning outcomes. Contrary findings were concluded in a study by Thunes and Sekse (2015) and Mwiinga, Maimbolwa and Muleya (2017), which found that midwifery students were dissatisfied with the clinical support received during clinical placement.

Registered midwives are deeply involved in supporting midwifery students during clinical placements. However, these supportive roles are voluntary and without any clinical training or support (Amod, Mkhize & Muraraneza 2021). The findings of this study showed that 51.7% of students received clinical support from registered midwives without a speciality qualification whilst only 28.3% of midwifery students received clinical support from senior midwives who are also midwifery clinical specialist. Likewise, a study by Simane-Netshisaula and Maputle (2021) concluded that many senior midwives such as midwifery clinical specialist, were unsupportive and excluded themselves from supervisory roles once they assume roles in clinical leadership. For midwifery students, the clinical support of all registered midwives during clinical placement is a stepping stone to success. Indeed, the challenges of clinical placements are expected but patient care is a definite priority and supporting midwifery students who are directly involved in patient care outcomes remain crucial.

Findings related to the perceived competence levels in midwifery requirements, across maternity care areas highlighted that midwifery students were incompetent in requirements such as performing pap smears; pelvic assessments; screening high-risk patients, performing and suturing an episiotomy; recognising physiological changes in the reproductive system; performing basic neonatal resuscitation; performing a first baby bath; and performing a stomach washout. These results revealed that midwifery students in the undergraduate programme are not ready for role-taking. Similarly, gaps in clinical support and competency levels of midwifery students in training was evident in a study by Malakooti et al. (2018) where midwifery students were incompetent in performing a pelvic assessment and neonatal resuscitation among other competencies. Mechanisms to bridge these competency gaps through clinical training and support programmes is widely recommended by Yigzaw et al. (2015), Kaphagawani and Useh (2018), Bradshaw et al. (2018), Feyissa et al. (2019), Sharifipour, Heydarpour and Salari (2020) and Stefaniak and Dmoch-Gajzlerska (2020).

## Conclusion

The clinical competence of midwifery students is highly reliant on the quality of clinical support they receive during clinical placement. Midwifery practitioners working in clinical placements have already adopted a supportive role in clinical teaching. Initiatives to strengthen this supportive role is necessary.

### Recommendations

In order to strengthen the clinical support of midwifery students, midwifery practitioners who voluntarily supervise midwifery students in practice should be empowered through formal training and support programmes. Measures to bridge the gaps identified in the competency levels of midwifery students post clinical placements should be carefully integrated into mentorship programmes during community service placements.

### Strengths and limitations

The outcomes of this study affirm the need to strengthen the clinical support and competency levels of midwifery students during clinical placement. This article contributes to the limited literature available on the clinical support of midwifery students in African countries. The recommendation from this article sets the ground for further research into related midwifery clinical support concerns.

The study findings should not be generalised as the study was restricted to one district within the province. The study did not evaluate the perceptions of midwifery practitioners regarding the clinical support they offered to the cohort of students in the same setting.

In this phase of the study, a quantitative research design was adopted. The researchers used two non-probability sampling techniques (purposive and convenient) to collect data from undergraduate midwifery students.
